# Dosimetric comparison of interstitial brachytherapy with multi-channel vaginal cylinder plans in patients with vaginal tumors

**DOI:** 10.1186/s13014-017-0821-0

**Published:** 2017-05-18

**Authors:** Lucas C. Mendez, Moti Paudel, Matt Wronski, Ananth Ravi, Lisa Barbera, Eric Leung

**Affiliations:** 1 Odette Cancer Centre, Sunnybrook Health Sciences Centre, Department of Radiation Oncology, University of Toronto, Toronto, Canada; 2 Odette Cancer Centre, Sunnybrook Health Sciences Centre, Department of Medical Physics, University of Toronto, Toronto, Canada

## Abstract

**Background:**

To evaluate the dosimetric performance of multi-channel vaginal cylinder (MCVC) against interstitial brachytherapy (ISBT) for the treatment of vaginal tumors.

**Methods:**

Vaginal tumors with extension of > 0.5 cm and ≤ 2 cm from the lateral vaginal wall and/or ≤ 1 cm in height above the vaginal vault were retrospectively selected from a ISBT registry trial database. The selected patients were treated with ISBT and targets included the intermediate (IRCTV) or high-risk (HRCTV) clinical target volumes. For technique comparison, a 35 mm MCVC was registered with the interstitial intra-vaginal cylinder. Bladder and rectum contours were transferred from the ISBT to the MCVC-BT plans. Vaginal mucosa was achieved by 3 mm uniform expansion from cylinder surface. Both the ISBT and MCVC-BT plans were optimized using the Inverse Planning Simulated Annealing optimization algorithm. After normalizing target D90 to 700 cGy, dose to organs at risk were measured and compared between ISBT and MCVC plans.

**Results:**

Six interstitial patient plans met the inclusion criteria for this study. Four patients had vaginal primaries and two recurrent cancers in the vagina. Lower doses to bladder and rectum were seen with ISBT plans. In half of the MCVC plans, the rectal dose met the recommended constraints. For plans in which the rectal constraint was not met, the target volumes were abutting the rectum and had a cranial-caudal length ≥ 5 cm. Dose to vaginal mucosa was lower in ISBT plans directed to the HRCTVs, although no difference was seen in circumferential IRCTVs.

**Conclusions:**

Overall, ISBT results in decreased dose to OARs as compared to MCVC. However, MCVC BT results in acceptable doses to OARs with possible improvement in vaginal doses for circumferential targets. Careful consideration to tumor geometry and location may help guide optimal techniques in vaginal tumor brachytherapy.

## Background

Brachytherapy (BT) plays an important role in the radiation treatment of vaginal tumors. Primary and recurrent cancers in the vagina are often treated with a brachytherapy boost after external beam radiation to the pelvis. Brachytherapy is able to deliver high-dose radiation to tumors due to the ability to place the source in close proximity or inside the target. The most common BT forms of treatment for vaginal tumors employ a single-channel vaginal cylinder (SCVC) and the interstitial BT (ISBT) techniques.

The American Brachytherapy Society has published guidelines on the treatment of vaginal cancers and suggests that ISBT is the preferred modality for lesions thicker than 5 mm [1]. For these tumors, SCVC brachytherapy treatment results in high doses to the vaginal mucosa and pelvic organs as compared to ISBT. However, interstitial brachytherapy treatment may be more invasive, resource intensive and inconvenient, as it usually requires hospital admission, general anesthesia and carries risks for pain and acute complications from the procedure.

Multi-channel vaginal cylinder (MCVC) BT has been developed to bridge the gap between SCVC and ISBT applicators [2]. With channels embedded in the periphery of the cylinder in addition to a central channel, MCVC can deliver more conformal doses than SCVC to regions in the vagina without the invasiveness of ISBT. Compared to SCVC, MCVC was shown to deliver decreased dose to OAR [3]. However, MCVC BT dosimetry has not been directly compared to ISBT.

The goal of this study is to evaluate the dosimetric performance of MCVC against ISBT in patients with vaginal tumors thicker than 5 mm, previously treated with ISBT.

## Methods

Patient plans from a single-institution interstitial brachytherapy approved by ethics committee registry trial were identified retrospectively for this study. Inclusion characteristics were recurrent or primary vaginal tumors with target volume depth of > 0.5 cm and ≤ 2 cm from the lateral vaginal wall and/or ≤ 1 cm height cranially from the vaginal vault. These tumors were treated with interstitial brachytherapy with a perineal template using post-implant CT-based planning.

### Treatment protocol

All patients were initially treated with external beam radiotherapy using the four-field box technique with a total dose of 45 Gy in 25 fractions followed by interstitial BT (ISBT) boost treatment. For ISBT, a perineal template *(Best Medical, Springfield, Virginia)* was used with a 2.2 cm diameter vaginal cylinder and a combination of intracavitary and interstitial 6 F 24 cm plastic catheters. Three or four BT fractions were delivered with a target dose of 700 cGy per fraction. Target volumes and organs at risk (OAR) were defined on CT images. Plans were produced using the Oncentra Brachy (Elekta AB, Stockholm, Sweden) treatment planning system.

### Registration and contouring

**Fig. 1:**
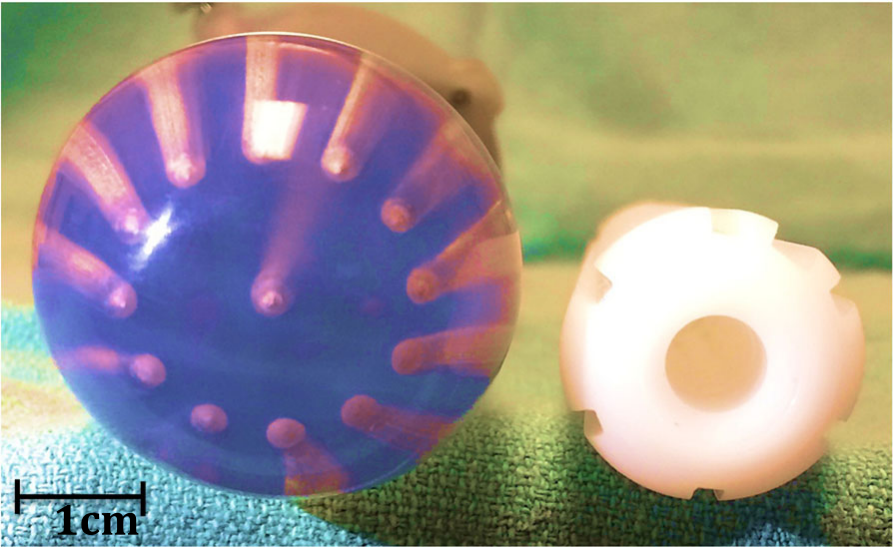
Multichannel vaginal cylinder with 35 mm in diameter and Syed-Neblett obturator with 22 mm

**Fig. 2:**
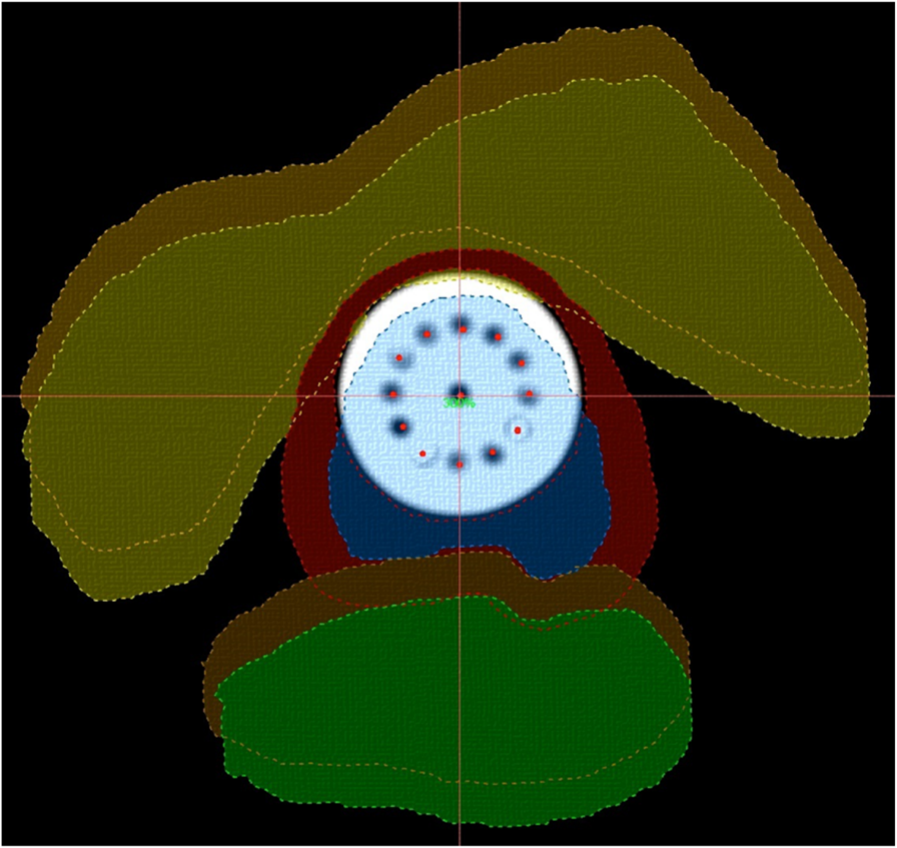
Translational shift of 6.5 mm to the bladder (*yellow* and *orange*) anteriorly and rectum (*brown* and *green*) posteriorly, with no tissue compression

For each ISBT treatment plan, a corresponding MCVC-BT plan was produced by rigid registration of the MCVC with the ISBT vaginal cylinder. This registration was performed using MIM (MIM Software Inc.) and allowed the MCVC-BT plans to inherit the OAR contours from the corresponding ISBT plans. A custom 3.5 cm diameter MCVC, with equally spaced twelve channels located at 9 mm radial distance from the central channel (Fig. 1), was chosen for comparison as this is the widest diameter cylinder that is commonly used and results in a favorable dose distribution. As the diameters of the ISBT vaginal cylinder and MCVC differed (Fig. 1), rigid registration was performed by aligning their central axes and tips. Bladder and rectum contours were transferred from the ISBT to the MCVC plans and translated in the anterior and posterior directions, respectively, to account for the larger MCVC diameter. The translation distance was equal to half the difference of the MCVC and ISBT cylinder diameters (0.65 cm). (Figure 2) Tissue compression was not taken into account. Target volumes were contoured on the MCVC-BT plans using clinical mark-up based on the clinical examination and target volume as seen on imaging.

Bladder and rectum were delineated in accordance to published guidelines [1]. As there is no current consensus on vaginal mucosa definition, it was defined by an isometric expansion of 2, 3 or 4 mm thickness from the cylinder surface excluding the cylinder and gross tumor. The HRCTV contour encompassed the gross disease at physical examination and imaging [4] and the IRCTV included the HRCTV plus possible sites of microscopic dissemination [5]. In advanced disease, the whole vaginal wall often was encompassed circumferentially by the IRCTV. In order to achieve a minimum dose of 65 Gy (EQD2) to the IRCTV, considering tumor’s α/β = 10, this volume was treated in the first two fractions alone followed by a more conformal boost to the HRCTV alone in the third ± fourth fractions. A minimum target dose of 75Gy (EQD2) was delivered to the HRCTV.

### Planning

Both the ISBT and MCVC-BT plans were optimized using the Inverse Planning Simulated Annealing (IPSA) optimization algorithm in Oncentra Brachy. Graphical dose optimization was subsequently carried out to maximize target volume conformality and minimize OAR doses. All plans were normalized such that 90% of the target volume received the same prescribed fractional dose of 700 cGy (D90% = 700 cGy). The highest fractional dose received by two cubic centimeters (D2cc) was evaluated for bladder and rectum. In accordance to ABS guidelines [1], OAR dose constraints were calculated using D2cc EQD2 total doses of 75Gy and 90Gy for rectum and bladder, respectively. For the purposes of this study, we analyzed a single fraction of treatment and therefore calculated the required OAR dose per fraction to be met to achieve target OAR constraints, assuming 4 brachytherapy fractions and 45 Gy in 25 fractions of external beam radiation. For rectum, D2cc per fraction was targeted to be under 496 cGy (α/β = 3) to meet a constraint of 75 Gy EQD2 and for bladder 630 cGy for 90 Gy EQD2. Vaginal mucosa D0.5 cc, D1cc and D2cc were evaluated. All dose calculation was based on the AAPM TG-43 formalism [6].

### Statistical analysis

Descriptive analyses comparing MCVC and interstitial plans were performed. Paired t-test was used for continuous variable comparison. A *p*-value <0.05 was considered statistically significant.

## Results

Six interstitial patient plans met the inclusion criteria for this study. Four were vaginal primaries (2 T1 and 2 T2) and two were recurrent cancers in the vagina. In four patients, two different volumes (HRCTV and IRCTV) were treated with interstitial brachytherapy in different fractions and analyzed. In one patient, the HRCTV was equal to the IRCTV and only one volume was treated. In another patient we analyzed only the HRCTV fraction as the IRCTV treatment was delivered with a different applicator (interstitial with tandem). In total, ten target volumes (5 HRCTVs and 5 IRCTVs) were dosimetrically compared between ISBT and MCVC plans.

Target volumes were variable in shape, size, thickness and proximity to adjacent OARs. All volumes had a maximum lateral thickness between 10 and 20 mm and a median volume of 37.5 ml (7.8–57.6 mL). Median cranial-caudal (CC) length was 50 mm (24–70 mm) (Table 1).

**Table 1:** Target characteristics of the 6 patients

CC Length (mm)	Circumferential shape	FL above cylinder tip (mm)	Max. Thickness (mm)	Max. Thickness Direction	Min. Bladder distance (mm)	Min. Rectum distance (mm)
42	Circumferential	5	12,4	Posterior-left	2	Abutting
68	Circumferential	5	17	Posterior-right	2	Abutting
50	Circumferential	10	20	Posterior-left	Abutting	Abutting
70	Circumferential	0	12	left	Abutting	Abutting
55	Circumferential	0	10	Anterior-right	Abutting	Abutting
30	1 to 7 h	7	12,8	left	2	Abutting
67	6 to 1 h	7	19	right	3	Abutting
24	3 to 8 h	6	15	posterior	8	Abutting
31	7 to 1 h	5	14	right	Abutting	9,4
50	Circumferential	2	11	Posterior-left	Abutting	Abutting

*FL* Free length

Overall, the bladder and rectum received higher doses in the MCVC plans than in the corresponding ISBT plans. The bladder D2cc mean values were 371 cGy (145–491 cGy) and 545 cGy (348–654 cGy) for interstitial BT and MCVC BT, respectively (*p* < 0.001). When HRCTV and IRCTV plans were analyzed separately, the bladder D2cc was also lower in ISBT plans as compared to MCVC (*p* < 0.05) (Table 2). However, in all MCVC plans except for one, the bladder dose did meet the target dose constraint of D2cc < 90 Gy EQD2 (≤630 cGy per fraction assuming 4 fractions of brachytherapy).

**Table 2:** Dosimetric comparison between ISBT and MCVC plans in HR-CTV and IR-CTV

	HRCTV (cGy)		*p*-value	IRCTV (cGy)		*p*-value
	ISBT	MCVC		ISBT	MCVC	
Bladder D2cc	338	556	0.0008	413	537	0.0001
Rectum D2cc	336	468	0.023	434	520	0.006
Vagina mucosa D0.5 cc	699	1134	0.003	1850	1534	0.09
Vagina mucosa D1cc	543	1009	0.0007	1448	1343	0.13
Vagina mucosa D2cc	383	864	0.0002	1161	1178	0.73

Rectal doses were also higher in MCVC plans. The mean rectal D2cc for ISBT and MCVC was 385 cGy (316–455 cGy) and 494 cGy (432–555 cGy), respectively (*p* < 0.009). When HRCTV and IRCTV plans were analyzed separately, the rectal D2cc was also lower in ISBT plans as compared to MCVC (*p* < 0.05) (Table 2). Recommended constraints of D2cc ≤ 75 Gy EQD2 (496 cGy per fraction × 4) was respected in half (5) of MCVC plans. For plans in which the rectal constraint was not met using MCVC, the target volumes were abutting the rectum and had a cranial-caudal (CC) length ≥ 5 cm. Also, in four out of five plans where the rectum constraint was not met, disease was above the cylinder tip and vaginal vault.

A better understanding of the vaginal mucosa dosimetry can be achieved by comparing HRCTV and IRCTV (circumferential) plans separately, since these volumes are conceptually different in terms of vaginal mucosa. As different vaginal dose parameters have been reported in previous publications, we used 3 dosimetric constraints (D0.5 cc, D1cc, D2cc) [7, 8]. In regards to HRCTV, ISBT plans had less dose to the vaginal mucosa than MCVC plans. Mean vagina mucosa D2cc, D1cc and D0.5 cc was 383 cGy vs. 864 cGy, 543 cGy vs. 1009 cGy and 699 cGy vs. 1134 cGy between ISBT and MCVC plans, respectively (*p* < 0.05). For IRCTV (circumferential), no significant differences in mean vaginal mucosa doses could be found between MCVC and ISBT plans. However, there was a trend for lower mean vaginal doses with MCVC. (Table 2).

There is no consensus in the literature regarding normal vaginal mucosa thickness, thus a sensitivity analysis using 2 and 4 mm mucosa thicknesses was performed. Plans treating the HRCTV targets volumes continued to show lower vaginal doses for the ISBT technique in both 2 and 4 mm mucosa thicknesses. However, MCVC plans targeting the IRCTV demontrasted a trend towards a reduced dose to the vaginal mucosa in comparison to ISBT. Table 3 reports the dose values in detail for different vaginal mucosa thicknesses in IRCTV plans.

**Table 3:** Dose relation between vaginal mucosa thickness and BT technique in IRCTV targets

Vaginal Mucosa	IR-CTV						
	ISBT (cGy)			MCVC (cGy)			
Thickness	D0.5 cc	D1cc	D2cc	D0.5 cc	D1cc	D2cc	*p*-value
2 mm	1767	1352	1061	1494	1289	1135	NS
3 mm	1850	1448	1161	1534	1343	1178	NS
4 mm	1915*	1501	1212	1537*	1370	1208	NS

*p = 0.06

A further analysis was done to evaluate vaginal mucosa doses taking into account tissue compression as a result of the wider cylinder of MCVC plans (Fig. 1). This comparison was done by comparing ISBT and MCVC plans with different vaginal mucosa thicknesses. ISBT plans with a 4 and 3 mm vaginal mucosa dose were compared to MCVC plans with a 3 and 2 mm mucosa thickness, respectively. In both comparisons, D0.5 cc was statistically higher for ISBT plans, while D1cc and D2cc were not different. Further details can be seen at Table 4.

**Table 4:** Exploratory analysis of vaginal mucosa dosimetry in ISBT, and MCVC plans after mucosa thinning

Comparison groups	Vaginal parameter	Dose		*p* Value
		ISBT (cGy)	MCVC (cGy)	
MCVC 2 mm vs ISBT 3 mm	D0.5 cc	1850	1494	0.04
	D1cc	1448	1289	NS
	D2cc	1161	1135	NS
MCVC 3 mm vs ISBT 4 mm	D0.5 cc	1915	1534	0.03
	D1cc	1501	1343	NS
	D2cc	1212	1178	NS

## Discussion

The relative advantages and disadvantages of MCVC versus ISBT have been generally based on assumption but these techniques have not been compared dosimetrically. This study compares MCVC and ISBT OAR doses in patients with vaginal tumors originally treated with interstitial BT using the ISBT CT planning image sets. For all the patients in this study, tumor thicknesses ranged from 10 to 20 mm. Overall, ISBT plans had consistently lower OAR doses as compared with MCVC plans when target D90 in each plan were adjusted to be equal. This was most apparent for non-circumferential volumes (HRCTV), where more dose sparing to bladder, rectum and vaginal mucosa was achieved with the interstitial technique. This is consistent with the highly conformal dose delivery of ISBT, where needles can be placed directly into tumor. Despite this, however, half of the MCVC plans did meet rectal constraints as per ABS guidelines when target coverage was adjusted to be equal to ISBT. MCVC plans where rectal constraints failed had 1) cranial-caudal lengths ≥ 5 cm, 2) tumor abutting the rectum and commonly 3) tumor cranial to the vaginal cylinder.

For circumferential volumes (IRCTV), ISBT also resulted in lower doses to the bladder and rectum as compared to MCVC, however no benefit was seen for the vaginal mucosa, differing from previous suggestions in the literature [9]. In fact, a trend towards lower vaginal mucosa dose was found in MCVC plans in this setting. This was consistent for vaginal mucosa D0.5 cc and D1cc constraints, where MCVC plans had mean doses that were lower by 316 cGy and 105 cGy respectively as compared to ISBT. This may relate to the ISBT cylinder geometry where the applicator is built with needle grooves on the cylinder surface (as opposed to channels within the cylinder as per MCVC), thus resulting in higher doses to the vaginal mucosa, which may be in direct proximity to loaded dwell positions. In addition, tissue compression from the vaginal cylinder was not accounted for in the MCVC plans, which may have led to an underestimation of MCVC’s sparing capabilities of the vaginal mucosa. It should also be noted that we delineated vaginal mucosa by subtracting out HRCTV, differing from previous publications that have included the target volume.

Limitations of this dosimetric study include a small sample size and the retrospective nature of this analysis. Furthermore, simulating the MCVC treatment on the interstitial brachytherapy CT data sets may be associated with some uncertainties. Firstly, a 3.5 cm cylinder was used for MCVC simulation to reflect the most commonly used cylinder size at our centre. The largest tolerated cylinder is typically chosen in order to obtain optimal apposition of the vaginal tissue with the cylinder surface and also to obtain a favorable dose distribution from the central channel. By translating the 3.5 cm cylinder to the interstitial CT dataset where the cylinder size is 2.2 cm a degree of tumor and target compression may be introduced and this was not reflected in our analysis. Also, the bladder and rectum were rigidly translated without accounting for minor organ changes that may result from the larger cylinder. Finally, target contours from the CT interstitial data set were used for planning of both techniques and contouring uncertainties may be present given the limitation in CT for soft tissue boundaries.

One potential strength of this study is the use of IPSA planning to eliminate bias associated with forward planning techniques.

## Conclusion

Interstitial BT was found to be dosimetrically superior to MCVC-BT for vaginal tumor plans. Cases where this is most apparent are in tumors 1) abutting the rectum, 2) ≥ 5 cm length or 3) above the cylinder. For circumferential tumors, the advantages of ISBT over MCVC are diminished as MCVC plans may result in reduced vaginal mucosa dose. Careful consideration of tumor location and geometry in addition to patient factors may help guide optimal treatment for patients with advanced vaginal disease.

## Abbreviations

BT: Brachytherapy
CC: Cranial-caudal
EQD2Gy: Equivalent dose in 2Gy fractions
HRCTV: High-risk clinical target volumes
IRCTV: Intermediate-risk clinical target volume
ISBT: Interstitial brachytherapy
MCVC: Multi-channel vaginal cylinder
OAR: Organs at risk
SCVC: Single-channel vaginal cylinder
